# COVID-19 Affects Serum Brain-Derived Neurotrophic Factor and Neurofilament Light Chain in Aged Men: Implications for Morbidity and Mortality

**DOI:** 10.3390/cells12040655

**Published:** 2023-02-17

**Authors:** Carla Petrella, Maria Antonella Zingaropoli, Flavio Maria Ceci, Patrizia Pasculli, Tiziana Latronico, Grazia Maria Liuzzi, Maria Rosa Ciardi, Antonio Angeloni, Evaristo Ettorre, Michela Menghi, Christian Barbato, Giampiero Ferraguti, Antonio Minni, Marco Fiore

**Affiliations:** 1 Institute of Biochemistry and Cell Biology (IBBC-CNR), Department of Sensory Organs, Sapienza University of Rome, 00185 Rome, Italy; 2 Department of Public Health and Infectious Diseases, Sapienza University of Rome, Viale del Policlinico 155, 00185 Rome, Italy; 3 Department of Experimental Medicine, Sapienza University of Rome, 00185 Rome, Italy; 4 Department of Biosciences, Biotechnologies and Biopharmaceutics, University of Bari “Aldo Moro”, 70121 Bari, Italy; 5 Department of Clinical, Internal Medicine, Anesthesiologic and Cardiovascular Sciences, Sapienza University of Rome, Viale del Policlinico 155, 00161 Rome, Italy; 6 Department of Maternal Infantile and Urological Sciences, Sapienza University of Rome, 00185 Rome, Italy; 7 Department of Sensory Organs, Sapienza University of Rome, 00185 Rome, Italy; 8 Division of Otolaryngology-Head and Neck Surgery, ASL Rieti-Sapienza University, Ospedale San Camillo de Lellis, Viale Kennedy, 02100 Rieti, Italy

**Keywords:** BDNF, NFL, NGF, MMP-2, MMP-9, NeuroCOVID, COVID-19, aging

## Abstract

Background and Methods: Severe COVID-19 is known to induce neurological damage (NeuroCOVID), mostly in aged individuals, by affecting brain-derived neurotrophic factor (BDNF), matrix metalloproteinases (MMP) 2 and 9 and the neurofilament light chain (NFL) pathways. Thus, the aim of this pilot study was to investigate BDNF, MMP-2, MMP-9, and NFL in the serum of aged men affected by COVID-19 at the beginning of the hospitalization period and characterized by different outcomes, i.e., attending a hospital ward or an intensive care unit (ICU) or with a fatal outcome. As a control group, we used a novelty of the study, unexposed age-matched men. We also correlated these findings with the routine blood parameters of the recruited individuals. Results: We found in COVID-19 individuals with severe or lethal outcomes disrupted serum BDNF, NFL, and MMP-2 presence and gross changes in ALT, GGT, LDH, IL-6, ferritin, and CRP. We also confirmed and extended previous data, using ROC analyses, showing that the ratio MMPs (2 and 9) versus BDNF and NFL might be a useful tool to predict a fatal COVID-19 outcome. Conclusions: Serum BDNF and NFL and/or their ratios with MMP-2 and MMP-9 could represent early predictors of NeuroCOVID in aged men.

## 1. Introduction

COVID-19 (coronavirus disease 19) is an acute respiratory syndrome induced by SARS-CoV-2 (severe acute respiratory syndrome coronavirus 2) [1,2,3,4,5]. The COVID-19 infection has expanded all over the world since December 2019 and has rapidly become one of the greatest challenges of our century, responsible for enormous medical, social, and economic problems. Even though the number of cases and deaths is decreasing from the initial stage, COVID-19 is still a leading cause of death worldwide [6].

One thing we certainly understand about SARS-CoV-2 is that the virus is mutating constantly. Since the beginning of the pandemic, a number of noticeable variants, including Alpha, Beta, Delta, and Omicron have been discovered [7,8,9].

COVID-19 clinical characteristics differ from asymptomatic presence to severe respiratory distress syndrome. The main COVID-19 clinical outcomes are dyspnea, cough, and fever [10,11]. It is known that asymptomatic patients played a significantly crucial role in the transmission of COVID-19 [12]. The progression towards a bad ending is due to several risk factors: a hyper-inflammation state elicited by our immune system, the incidence of comorbidities, the advanced age of the affected individuals, and a higher risk of thrombosis [13,14,15,16].

During the paroxysm inflammation period, cytokine overproduction and the disrupted innate immune response may provoke parenchymal damage in vital organs and consequent failure in different tissues and organs [17]. COVID-19 is known to cause serious damage and greater mortality in aged individuals. However, COVID-19-associated casualties have also been observed in children, adolescents, and young adults [14,18,19]. Indeed, several pieces of evidence disclosed the occurrence of comorbidities, such as diabetes, cancer, and cardiovascular diseases that may influence the prognosis in older individuals with COVID-19 disease [15].

The potential target organs of COVID-19 also include the brain and, more generally, the central and peripheral nervous system (NeuroCOVID), which may be affected during and after the disease [7,8,9]. Neurotrophins are polypeptides recognized to control neuronal survival, growth, and morphology during development and in the adult brain [20,21,22]; they also regulate strategic nerve cell functions such as synaptogenesis, excitability, and brain aging [23,24]. The NGF (nerve growth factor), BDNF (brain-derived neurotrophic factor), neurotrophin-3 (NT-3), NT-4, NT-5, NT-6, and NT-7 are the important neurotrophins with neuroprotective and neurotrophic activities in several nerve cell populations, both in the peripheral and central nervous system. In physiological and/or pathological circumstances, NGF and BDNF, the most studied neurotrophins, influence not only neuronal cells but, likewise, play subtle functions in the endocrine, immune, and cardiometabolic systems to control biological homeostasis [25,26,27,28,29,30,31], crucially regulating inflammatory processes [32,33].

As for the relationship between NeuroCOVID and NGF/BDNF, to date, only a few papers discussed their potential role in this disease. Previous studies debated the role of NGF in pulmonary pathologies, alluding to the possibility of considering the NGF signaling as a potential diagnostic/therapeutic target in SARS-CoV-2-induced pulmonary complications [34], contributing to the antibody production in convalescent COVID-19 [35]. Other investigations proposed that serum BDNF content and the BDNF/adiponectin ratio may serve as predictors of a worsened prognosis in COVID-19, especially for adult male patients [36], with BDNF also playing a subtle role in the neurological and mental outcomes of COVID-19 [37,38].

Our previous data on children and adolescents [18] showing the long-lasting effects of the infection (long-COVID-19) revealed that NGF serum content was lower in post-infected-COVID-19 individuals when compared to healthy controls; interestingly, BDNF levels were found to be higher compared to healthy individuals only in post-infected-COVID-19 symptomatic and future long-COVID-19 girls, leaving the BDNF levels unchanged in asymptomatic individuals when compared to controls [18]. In a recent study, increased NGF and BDNF levels were quantified in saliva and serum during the acute phase of SARS-CoV-2 infection in hospitalized patients, but reduced levels were observed in the remission phase, never matching the baseline values [39]. Other data showed that the matrix metalloproteinase-9 (MMP-9)/BDNF ratio predicts more severe COVID-19 outcomes [40] as MMPs may contribute to the tissue damage induced by COVID-19 [41] as well as to the alteration of blood–brain barrier integrity [42]. NeuroCOVID is also characterized by neurodegeneration and elevations in the neurofilament light chain (NFL) in the blood, which may represent a useful tool to investigate the disease severity in COVID-19-affected individuals [43,44].

Thus, the aim of this pilot study was to investigate the NGF, BDNF, MMP-2, MMP-9, and NFL in the serum of aged men affected by COVID-19 at the beginning of the hospitalization period and characterized by different outcomes, i.e., a *hospital ward* group vs. an intensive care unit (*ICU*) group vs. a fatal outcome (*deceased*) group. As a *control* group we used, as the main novelty of the study, unexposed age-matched men. We predicted that BDNF and NFL presence in COVID-19 in aged individuals might be associated with severe morbidity and fatal outcomes. We also predicted that the ratios of BDNF and NFL with MMP-2 and MMP-9 could be considered early predictors of COVID-19 mortality. Furthermore, we correlated these findings with some routine laboratory analyses performed on COVID-19 patients such as amylase, lipase, aspartate transaminase (AST), alanine transaminase (ALT), gamma-glutamyl transferase (GGT), lactate dehydrogenase (LDH), myoglobin (MGB), total creatine kinase (CK), isoenzyme MB of creatine kinase (CK-MB), high sensitivity troponin-T (TnT), interleukin-6 (IL-6), ferritin, C-reactive protein (CRP), plateletcrit (PCT), D-dimer, platelet count (PLT), prothrombin time (PT), the international normalized ratio (INR), the activated partial thromboplastin time (aPTT), fibrinogen (FBG), and white blood cell (WBC) number.

## 2. Materials and Methods

### 2.1. Participant Selection and Study Design

Blood samples were collected from 30 COVID-19-infected male aged patients (randomly chosen in a larger cohort group) at the *beginning* of their COVID-19 hospital ward permanence in the Sapienza University Hospital “Policlinico Umberto I” in Rome, Italy, between May 2020 and July 2020. At that time, vaccines were not yet available. Accordingly, the COVID-19 individuals were divided into three groups based on their outcomes. The first group included patients who entered the hospital wards and survived (*hospital ward* group, *n* = 10; age range in years 74–90). The second group included patients who after the COVID-19 hospital ward attended the COVID-19 intensive care unit and survived (*ICU* group, *n* = 10; age range in years 67–93). The third group included patients who after the COVID-19 hospital ward attended the COVID-19 intensive care unit but did not survive (*deceased* group, *n* = 10; age range in years 70–95). The real-time reverse-transcription polymerase chain reaction (RT-PCR) test on nasopharyngeal swab samples was used to identify SARS-CoV-2-infected individuals.

As controls, we used the blood samples of aged male healthy volunteers recruited at the Sapienza University blood donor group (*control* group, *n* = 10; age range in years 70–85). The main exclusion criteria to avoid any bias in the selection of controls belonging at the beginning of the recruitment to a much larger cohort included other ongoing pathologies and previous inflammatory, endocrine, and autoimmune disorders. We also excluded aged men with diagnosed cardiovascular pathologies that could have biased inflammatory analyses, and previous use of drugs or chemicals that can alter the serum levels of inflammation markers, such as antidepressants, anti-inflammatories, and immunosuppressants. The University Hospital ethical committee approved the study (Ref. 6536), and all study procedures followed the Helsinki Declaration of 1975, as revised in 1983, for human rights and experimentation.

### 2.2. Blood Withdrawal

According to methods previously described [45], peripheral blood samples of 5 mL were taken from each participant, collected in BD Vacutainer™ serum separation tubes, and centrifuged at 3000 rpm for 15 min to separate serum. Serum was then stored at −80 °C.

### 2.3. Data Collection

We extracted information on demographic characteristics, laboratory analytical results, and symptoms for each eligible patient. The results of the laboratory tests were collected during hospitalization at the Sapienza University Hospital “Policlinico Umberto I” in Rome. Laboratory analytical results included albumin, glycemia, pancreatic biomarkers (amylase and lipase), liver biomarkers (AST, ALT, and GGT), LDH, cardiac biomarkers (CK, MGB, and TnT), inflammatory biomarkers (IL-6, ferritin, CRP, and PCT), WBC, and coagulation profiles (PLT, PT, INR, PTT, FBG, and D-dimer). Data extracted from the patient medical records were related to cardiovascular diseases, heart failure, diabetes, chronic obstructive pulmonary disease, chronic hepatitis, chronic renal failure, solid neoplasm, leukemia, and possible transplants (Table 1). Recruited patients underwent the pharmacological treatments available at that time, such as antibiotics, anti-inflammatory and antipyretic drugs, and heparin.

### 2.4. NGF and BDNF Serum Evaluation

NGF (Cat. No. DY256) and BDNF (Cat. No. DY248) were measured using a sandwich enzyme-linked immunosorbent assay (ELISA) kit (R&D Systems, Minneapolis, MN, USA), according to the protocols provided by the manufacturer [46]. Serum samples were diluted 2- and 100-fold with PBS for the detection of NGF and BDNF, respectively. The colorimetric reaction product was measured at 450 nm using a microplate reader (Dynatech MR 5000, PBI International, Washington, DC, USA). Data are represented as pg/mL, and all assays were performed in duplicate, which was averaged for statistical comparison [47].

### 2.5. NFL Serum Evaluation

As previously described [42,48], the evaluation of NFL levels in collected samples was assessed using the Simple Plex^TM^ Ella assay (ProteinSimple, San Jose, CA, USA) on Ella^TM^ microfluidic system (Bio-Techne, Minneapolis, MN, USA) according to the manufacturer’s instructions. Ella^TM^ was calibrated using the in-cartridge factory standard curve. The limit of detection of NFL was 1.09 pg/mL, calculated by adding three standard deviations to the mean background signal determined from multiple runs.

### 2.6. MMP-2 and MMP-9 Serum Evaluation Using Zymography

As previously described [49], MMP-2 and MMP-9 serum activities were detected using zymography as white bands of digestion on the blue background of the gel and were identified by co-localization on the zymogram with human MMP-2 or MMP-9 standards (ALEXIS Biochemicals, San Diego, CA, USA). Quantitation of MMP-2 and MMP-9 serum activities were performed using computerized image analysis (Image Master 1D, Pharmacia Biotech, Buckinghamshire, UK) through one-dimensional scanning densitometry (Ultroscan XL, Pharmacia Biotech). MMP serum activities were expressed as optical density (OD) × mm^2^, representing the scanning area under the curves, which considers both brightness and width of the substrate lysis zone.

### 2.7. Laboratory Examination

The patient’s peripheral blood was collected in BD vacutainer^®^ tubes for blood testing at the entrance of the *hospital ward* group and at the beginning of the intensive care period for both the *ICU* and *deceased* patient groups. The additives present in vacutainers were EDTA or sodium citrate as anticoagulants and separating gel from serum. Coagulation parameters were analyzed using a BCS XP System automatic hemostasis analyzer (Siemens Healthcare, Germany). Immunoturbidimetric assay and modified Claus method were used, respectively, for D-dimer (reference range: 50–420 μg/L) and fibrinogen (reference range: 1.5–4 g/L); the interassay coefficient of variation (CV), tested in normal and pathological control sera, was, respectively, between 2.2 and 4.3% and between 1.6 and 3.4%. PT (reference range: 11–16 s), INR (reference range: 0.8–1.2), and APTT (reference range: 25–35 s) were determined coagulometrically; the interassay coefficient of variation (CV), tested in normal and pathological samples, was between 1.5 and 2.2% for PT and between 0.3 and 2.8% for aPTT. The number of platelets (reference range: 150–450 cells/μL) and WBC (reference range: 4400–11,300 cells/L) was determined using ADVIA 2120i Hematology System (Siemens Healthcare, Germany). Tissue biomarkers included albumin (reference range: 35–55 g/L), glucose (reference range: 70.3–100.9 mg/dL), amylase (reference range: 28–100 U/L), lipase (reference range: 13–60 U/L), AST (reference range: 9–45 U/L), ALT (reference range: 10–40 U/L), GGT (reference range: 8–61 U/L), CK (reference range: 20–200 U/L), ferritin (reference range: male 30–400 μg/L; female 15–150 μg/L), CRP (reference range: 100–6000 μg/L), and LDH (reference range: 135–225 U/L) that were measured using standard colorimetric and enzymatic method on a Cobas C 501 analyzer with reagents supplied by Roche Diagnostics GmbH (Mannheim, Germany). CV was, respectively, 0.9% at serum albumin of 51.3 g/L, 1.1% at serum glucose of 95.1 mg/dL, 2.4% at a serum amylase of 35 U/L, 1.4% at a serum lipase of 48 U/L, 2.3% at a serum AST of 30 U/L, 2.6% at a serum ALT of 24 U/L, 3.2% at a serum GGT of 46.8 U/L, 3.2% at a serum CK of 18.7 U/L, 2.8% at serum ferritin of 26.1 μg/L, 1.3% at a serum CRP of 39.9 mg/L, and 2.7% at a serum LDH of 124 U/L. CK-MB (reference range: until 4.94 μg/L), MGB (reference range: 28–72 μg/L), TnT (reference range: until 0.014 μg/L), IL-6 (reference range: 1.5–7 pg/mL), and PCT (reference range: 0.02–0.064 ng/mL) were measured on a Cobas E 601 analyzer, using sandwich immunological methods with reagents supplied by Roche Diagnostics GmbH (Mannheim, Germany). The interassay CV was, respectively, 1.4% at a serum CK-MB of 5.34 ng/mL, 1.9% at a serum MGB of 60.5 ng/mL, 2.7% at a serum TnT of 0.017 μg/L, 3.1% at a serum IL-6 of 12.1 pg/mL, and 8.7% at a serum PCT of 0.08 ng/mL.

### 2.8. Statistical Analysis

According to methods previously described [50,51], data were analyzed to assess normality using Pearson’s chi-square test. The analysis of variance (ANOVA) was used to analyze the laboratory parameters (*hospital ward* vs. *ICU* vs. *deceased* vs. *control* groups). Post-hoc comparisons were carried out using Tukey’s HSD test [52]. The Spearman correlation test was used to investigate the correlation between the laboratory data and the age of the patients. A receiver operating characteristic (ROC) analysis was performed to measure the diagnostic/predictive accuracy of the ratios MMP-x/NGF-BDNF-NFL. Only area under the curve (AUC) values >9 were considered as predictive scores.

## 3. Results

**Figure 1:**
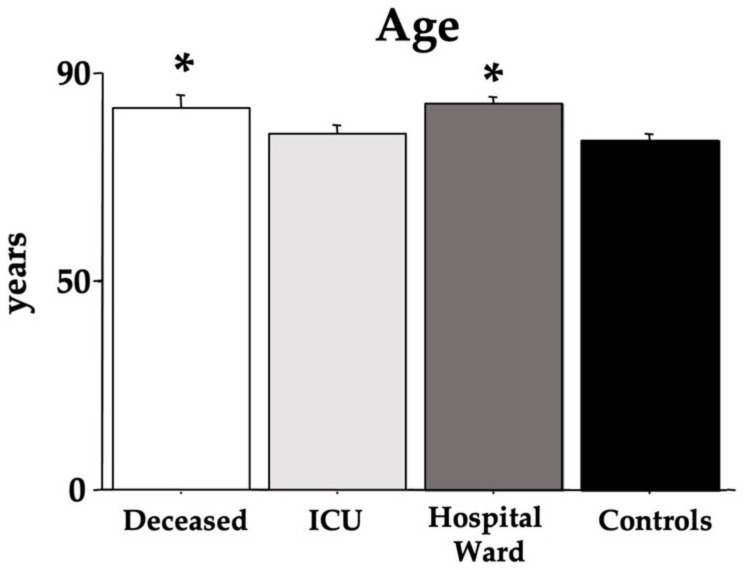
Age of the COVID-19 patients. The error bars indicate pooled standard error means (SEM) derived from the appropriate error mean square in the ANOVA. The asterisks (*p* < 0.05) indicate post hoc differences with controls.

The age of the recruited COVID-19 individuals and controls is shown in Figure 1. ANOVA did evidence an effect of morbidity. Indeed, both the *deceased* and *hospital ward* groups had higher ages compared to the *ICU* and *control* groups [F(3,36) = 4.72, *p* < 0.01; see the post hoc differences in the picture].

The clinical characteristics of COVID-19 patients according to their medical records are shown in Table 1. As expected, in the deceased group, the presence of previous cardiovascular diseases and diabetes was observed. Vital signs (heart rate, temperature, blood pressure, respiratory rate, and oxygen saturation) were not considered and correlated with the blood parameters as these findings were not recorded for all patients at the moment of blood withdrawal.

**Figure 2:**
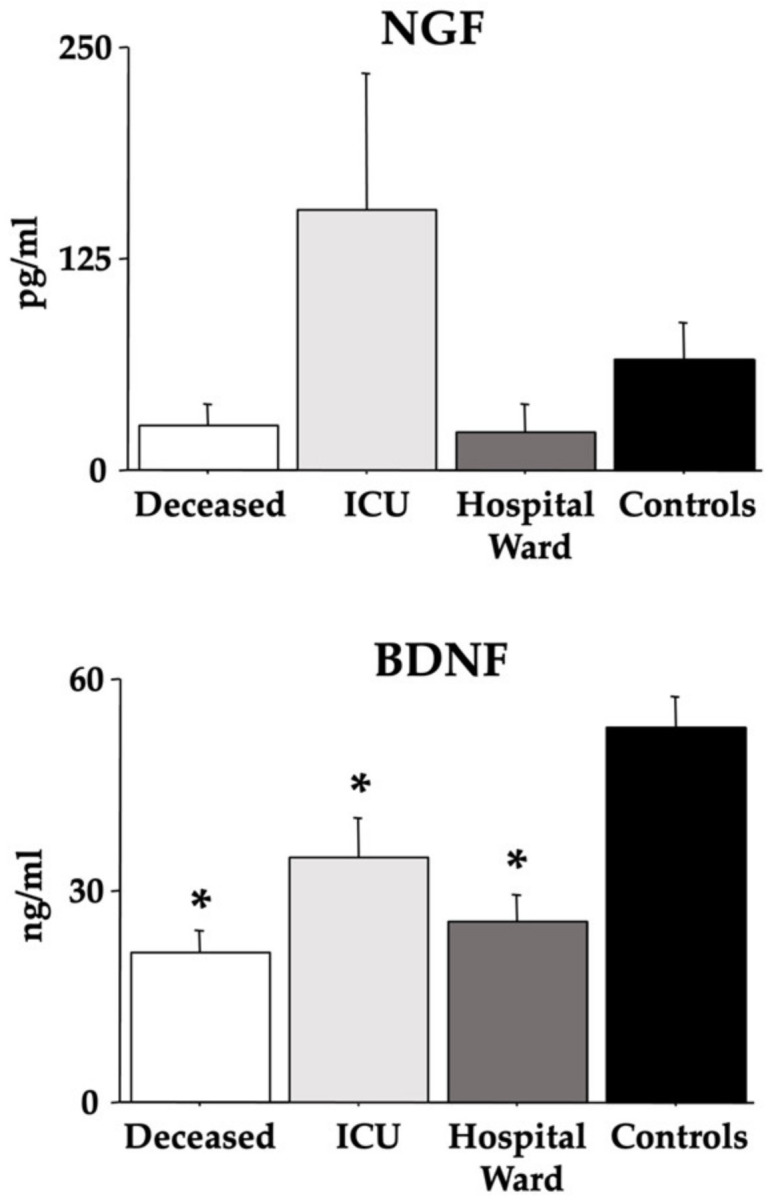
Serum NGF and BDNF in COVID-19 patients. The error bars indicate pooled standard error means (SEM) derived from the appropriate error mean square in the ANOVA. The asterisks (*p* < 0.05) indicate post hoc differences with controls.

Figure 2 shows the data on NGF and BDNF. ANOVA NGF data did not disclose differences between the groups [F(3,36) = 2.01, *p* = 0.12]. However, BDNF values were significantly lower in all COVID-19 groups (*deceased*, *ICU*, and *hospital ward*) compared to *control* individuals [F(3,36) = 11.34, *p* < 0.01; see the post hoc differences in the picture].

**Figure 3:**
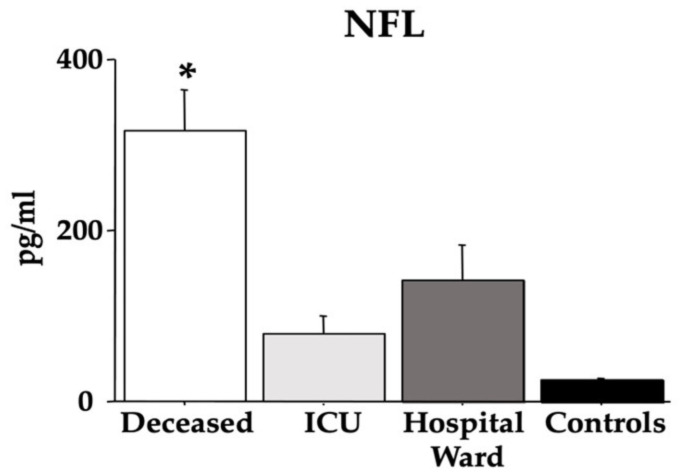
Serum NFL in COVID-19 patients. The error bars indicate pooled standard error means (SEM) derived from the appropriate error mean square in the ANOVA. The asterisk (*p* < 0.05) indicates post hoc differences between the *deceased* group and *ICU*, *hospital ward*, and *control* individuals.

Figure 3 reports the results of the NFL. Quite interestingly, the *deceased* group displayed the highest values compared with the other groups of individuals (*control*, *ICU*, and *hospital ward*) [F(3,36) = 14.94, *p* < 0.01; see the post hoc differences in the picture].

**Figure 4:**
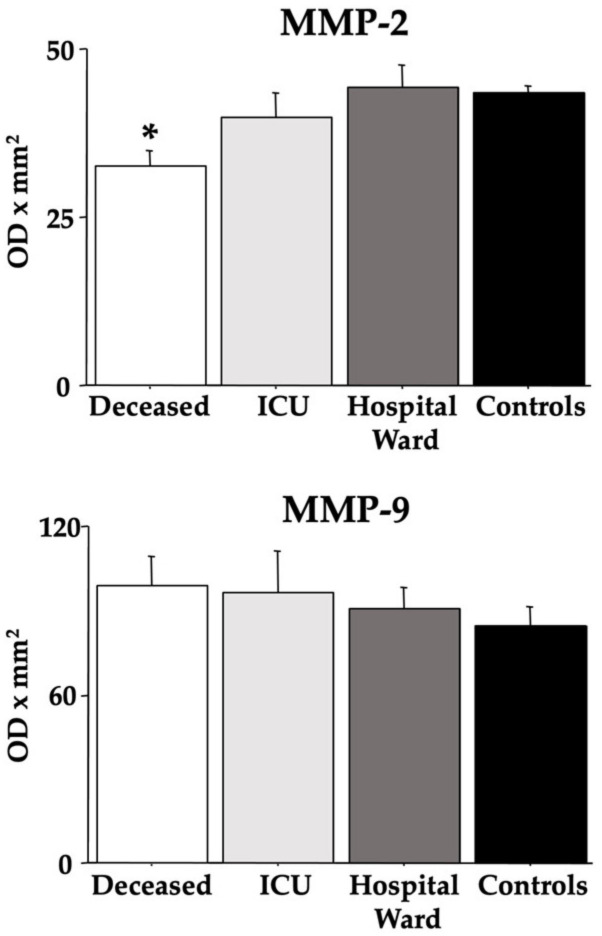
Serum MMP-2 and MMP-9 in COVID-19 patients. The error bars indicate pooled standard error means (SEM) derived from the appropriate error mean square in the ANOVA. The asterisk (*p* < 0.05) indicates post hoc differences with controls.

Figure 4 presents the MMP-2 and MMP-9 data. ANOVA revealed low MMP-2 values in the *deceased* group compared to both the *control* and *hospital ward* groups [F(3,36) = 3.74, *p* = 0.019; see the post hoc differences in the picture], but for the MMP-9 data, there were no differences between groups [F(3,36) = 0.38, *p* = 0.76].

**Figure 5:**
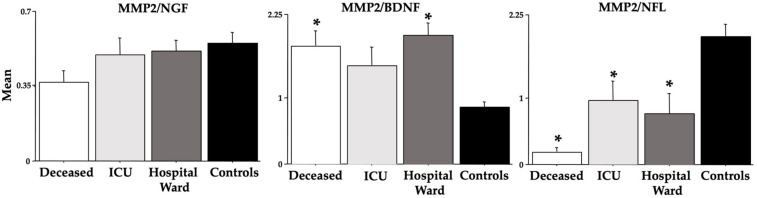
MMP-2 ratios vs. NGF, BDNF, and NFL in COVID-19 patients. The error bars indicate pooled standard error means (SEM) derived from the appropriate error mean square in the ANOVA. The asterisk (*p* < 0.05) indicates post hoc differences with controls.

**Figure 6:**
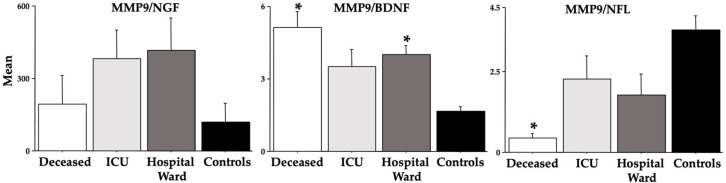
MMP-9 ratios vs. NGF, BDNF, and NFL in COVID-19 patients. The error bars indicate pooled standard error means (SEM) derived from the appropriate error mean square in the ANOVA. The asterisk (*p* < 0.05) indicates post hoc differences with controls.

Figure 5 and Figure 6 show the ratios between the analyzed MMPs and NGF, BDNF, and NFL. Indeed, for the *deceased* group, the MMPs/BDNF ratios were higher [F(3,36) = 7.64 (MMP-9), 5.10 (MMP-2), ps < 0.01; see post hoc differences in the figure], but the MMPs/NFL ratios were lower [F(3,36) = 6.55 (MMP-9), 9.60 (MMP-2), ps < 0.01; see post hoc differences in the figure].

Table 2 shows the AUC scores of the ROC curves for the MMP-2/NGF, MMP-2/BDNF, MMP-2/NFL, MMP-9/NGF, MMP-9/BDNF, and MMP-9/NFL ratios. The highest scores (in bold) were disclosed in the *deceased* group, in particular for the MMP-2/BDNF, MMP-2/NFL, MMP-9/BDNF, and MMP-9/NFL ratios.

Table 3 shows the Spearman correlations between age and NGF, BDNF, NFL, MMP-9, and MMP-2). No correlations were disclosed for the COVID-19 individuals (all groups or in the *deceased* group), but a positive correlation with age was revealed for NFL in *control* individuals.

Table 4 shows the ANOVA data on amylase, lipase, AST, ALT, GGT, LDH, MGB, CK, CK-MB, TnT, IL-6, ferritin, CRP, PCT, D-dimer, PLT, PT, INR, aPTT, FBG, and WBC. Data revealed the expected increasing effect of COVID-19 for ALT, GGT, LDH, ferritin, and CRP in the *deceased* group and for IL-6 in the *ICU* individuals. An elevation in ALT values was also observed in the *hospital ward* group compared to *controls*.

Table 5 shows, for the individuals of the deceased group, the Spearman correlations between the blood parameters and NGF/BDNF/NFL/MMP-2/MMP-9, and the positive values are displayed in bold. Indeed, MGB correlates positively with BDNF, CK with NFL, and MMP-2 with different trends, and FBG correlates negatively with BDNF, MMP-2, and MMP-9. No correlations were disclosed for NGF.

## 4. Discussion

This is the first study to demonstrate that, by using healthy age-matched controls, severe or lethal COVID-19 in aged, hospitalized men heavily disrupted BDNF, NFL, and MMP-2 presence in the serum of infected individuals, whereas no differences between groups were found for NGF and MMP-9. We also confirmed and extended previous data [40] showing that the ratios of MMPs (2 and 9) versus BDNF and NFL might be a useful tool to predict a fatal COVID-19 outcome. As for the routine laboratory data, as previously shown in many studies, we found gross changes in ALT, GGT, LDH, IL-6, ferritin, and CRP are a consequence of severe COVID-19 in aged men.

Neurotrophins, such as NGF and BDNF, have multiple roles in different settings, including neuronal development and survival and function in both the central and peripheral nervous systems from early stages [53,54,55]. Alterations in NGF and/or BDNF have been associated with several pathologic manifestations, including behavioral aberrations, cognitive deficiencies, tumorigenesis, obesity, and epilepsy, as well as muscle-skeletal, inflammatory, and pain sensitivity diseases [56,57]. In previous studies, a correlation was found in COVID-19 patients between low BDNF presence and neurological or cognitive decline [58,59]. These findings are in line with the present study showing low BDNF in all COVID-19 individuals with no differences related to their outcome. However, another investigation revealed increased NGF and BDNF in saliva and serum during the acute COVID-19 phase, but reduced levels were observed 6 months after the acute phase [39].

Regarding the serum NFL, we found potentiated values only in individuals with a lethal outcome. Indeed, blood NFL has been proposed to act as an assessment of COVID-19 severity in hospitalized patients and a NeuroCOVID biomarker. It has been previously shown that NFL concentrations in the plasma of COVID-19 within 5 days of hospital admission were elevated [43]. This NFL elevation was associated with worse clinical outcomes, including the need for intensive care, prolonged hospitalization, mechanical ventilation, and higher functional disability at the discharge [43]. In a NeuroCOVID study, infected patients with severe neurological manifestations (encephalopathy, meningoencephalitis, disrupted movement disorder, or stroke) had increased NFL in the cerebrospinal fluid. Another investigation speculated that serum NFL could be associated with severe outcomes in geriatric patients [60].

As for the modifications we found in MMP-2 and the absence of alterations in MMP-9, it is well known that COVID-19 predominantly affected the respiratory tract leading to acute lung failure as the most severe outcome. It is also well established that pulmonary infection may be associated with hyper-inflammation and tissue damage. The MMPs may have a role in lung damage in many pathological situations leading also to the release of bioactive molecules with inflammatory action [61,62,63]. Previous COVID-19 findings showed elevated MMP-2 and MMP-9 levels in hospitalized patients [62,64]. Upregulated MMP-9 genes in COVID-19 patients, together with an increased level of MMP-9, was associated with a risk of respiratory failure [65]. Other researchers, however, found low levels of MMP-2 [66]. Changes in MMPs in severe COVID-19 patients may play subtle roles in disrupting the alveolar–capillary barrier, drawing inflammatory cells and injuring the lung parenchyma [67]. Indeed, the degradation in connexins, elastin, and extracellular collagen by MMPs might be potentiated by the stiffer collagen rapid replacement, which could induce fibrosis and other COVID-19 problems [67].

We also investigated the ratios MMP-2/-9 vs. NGF/BDNF/NFL because it was proposed as a novel method to predict COVID-19 morbidity and mortality [40]. We found significant differences due to COVID-19 disease severity for both BDNF and NFL but not for NGF in the ratios with MMP-2 or MMP-9 (see Figure 5 and Figure 6). In particular, high ratios which were severity-related were found between MMP-2-9 and BDNF, but low ratios which were severity-related were evidenced between MMP-2/-9 and NFL. In a study by Savic et al. [40], although no modifications in BDNF were disclosed, the MMP-9/BDNF ratio was significantly depleted in severely and critically COVID-19 patients, recommending this ratio as a predictor of COVID-19 severity. Based on the ratio data of the present research, we extend to MMP-2 and NFL as markers of COVID-19 morbidity and mortality as also shown by the AUC data of the ROC curves.

As for the laboratory data, we discovered in patients of the *deceased* group potentiations in ALT, GGT, LDH, ferritin, and CRP. In our previous investigation carried out always at the Sapienza University Hospital “Policlinico Umberto I” [1], we studied early blood routine parameters as crucial in predicting biomarkers of COVID-19 morbidity and mortality. We have demonstrated that already at admission in the emergency section, COVID-19 individuals who underwent a lethal outcome displayed higher concentrations of AST, ALT, MGB, LDH, ferritin, CRP, and D-dimer compared to patients who were discharged from the emergency room, confirming and extending the huge plethora of COVID-19 laboratory data available in the scientific literature [68,69,70]. Indeed, clinical laboratory professionals are continuously searching for reliable parameters associated with SARS-CoV_(x)_ diseases for the proper clinical cure as blood biomarkers are commonly used to recognize, during the early stages of disease, patients at a higher risk of developing severe outcomes [1]. Spearman correlations scores (Table 5) underline the MGB and FBG ANOVA findings for the individuals of the *deceased* group.

## 5. Conclusions

The strength and novelty of this investigation were to study neuroinflammatory biomarkers in COVID-19-aged men according to their outcomes during the pandemic paroxysm wave and in absence of vaccines and compare these data with the findings obtained in healthy age-matched men. Of course, the absence of data on women and the relatively small number of the recruited individuals in this research could be putative limitations of the research. However, its strength depends on the choice of restricted recruitment guidelines.

Furthermore, with the purpose of showing other main potential COVID-19 biomarkers [1,50], this pilot study provides additional information aimed at disclosing further biomolecular events consequent to SARS-CoV-2 infection. Particularly, serum BDNF and NFL could represent a new tool as early predictors of NeuroCOVID effects in aged men associated with their ratios with MMP-2 and MMP-9. However, further investigations on the molecular and cellular link between neuroinflammation and neurodegeneration in COVID-19 are needed.

## Figures and Tables

**Table 1:** Clinical characteristics of COVID-19 patients according to their medical records.

	*Hospital Ward*	*ICU*	*Deceased*
**Symptoms**	*n* = 10	*n* = 10	*n* = 10
Fever	6	5	4
Cough	5	1	2
Dyspnea	2	8	7
Nausea	3	1	0
Dysgeusia	1	0	0
Anosmia	1	1	0
Arthralgia	0	0	0
**Diseases**			
Cardiovascular Diseases	6	4	2
Heart Failure	2	2	2
Diabetes	2	3	4
Chronic Renal Failure	1	0	0
COPD	1	1	0
Chronic Liver Diseases	1	1	1
Solid Neoplasm	1	1	0
Hematological Neoplasm	1	0	0
Transplant	0	0	0

**Table 2:** AUC scores for the MMP-2/NGF, MMP-2/BDNF, MMP-2/NFL, MMP-9/NGF, MMP-9/BDNF, and MMP-9/NFL ratios. The highest scores (in bold) were disclosed in the *deceased* group.

	*Deceased* vs. *Controls*		*ICU* vs. *Controls*	
	The Area under the Curve (AUC)	95% CI for AUC	The Area under the Curve (AUC)	95% CI for AUC
**MMP-2/NGF**	0.85	0.675–1	0.620	0.358–0.882
**MMP-2/BDNF**	**0.940**	0.848–1	0.755	0.499–1
**MMP-2/NFL**	**1**	1–1	0.835	0.620–1
**MMP-9/NGF**	0.710	0.467–0.953	0.620	0.333–0.907
**MMP-9/BDNF**	**0.990**	0.962–1	0.755	0.499–1
**MMP-9/NFL**	**1**	1–1	0.835	0.62–1

**Table 3:** Spearman correlation values for the age parameters in all groups. Significant scores are shown in bold.

	*Controls*			*All COVID-19 Groups*			*Deceased*		
	SSD	Rho	*p*-Value	SSD	Rho	*p*-Value	SSD	Rho	*p*-Value
**NGF**	267.000	−0.618	0.063	4229.500	0.059	0.750	150.500	0.088	0.792
**BDNF**	234.500	−0.421	0.206	6018.500	−0.338	0.0680	148.500	0.100	0.7642
**NFL**	42.500	0.742	**0.025**	3067.000	0.318	0.0871	160.500	0.027	0.082
**MMP-2**	96.500	0.415	0.213	4338.000	0.035	0.850	127.500	0.227	0.495
**MMP-9**	165.500	−0.003	0.992	4264.000	0.051	0.782	146.500	0.112	0.7366

**Table 4:** ANOVA data for some routine blood parameters in all groups. Significant scores are shown in bold. The asterisks indicate significant differences with *controls*.

	*Deceased*	*ICU*	*Hospital Ward*	*Controls*	*p*	*F, dF*
**Amylase** in U/L	99.6 ± 36.74	91.20 ± 9.77	51.10 ± 5.30	62.30 ± 6.61	0.25	(3,36) = 1.40
**Lipase** in U/L	50.80 ± 9.00	42.50 ± 5.82	35.20 ± 5.52	34.60 ± 3.51	0.24	(3,36) = 1.46
**AST** in U/L	39.80 ± 12.36	22.80 ± 3.38	30.60 ± 4.47	17.25 ± 1.25	0.16	(3,36) = 1.80
**ALT** in U/L	36.70 ± 11.36 *	29.5 ± 5.25	38.90 ± 8.11 *	6.12 ± 0.51	**0.033**	(3,36) = 3.32
**GGT** in U/L	73.60 ± 17.39 *	46.90 ± 11.09	26.40 ± 5.34	17.20 ± 2.32	**0.003**	(3,36) = 5.44
**LDH** in U/L	367.00 ± 80.37 *	242.90 ± 26.18	245.70 ± 27.33	133.40 ± 6.01	**0.008**	(3,36) = 4.56
**MGB** in µg/L	54.20 ± 7.66	77.59 ± 23.31	50.50 ± 8.69	57.36 ± 7.53	0.50	(3,36) = 0.78
**CK** in U/L	103.20 ± 27.74	100.80 ± 28.44	102.80 ± 46.55	84.70 ± 8.58	0.96	(3,36) = 0.08
**CK-MB**	3.39 ± 0.34	2.42 ± 0.20	3.12 ± 0.18	2.77 ± 0.25	0.062	(3,36) = 2.66
**TnT** in µg/L	0.039 ± 0.018	0.042 ± 0.017	0.021 ± 0.013	0.013 ± 0.002	0.39	(3,36) = 1.03
**IL-6** in pg/L	65.54 ± 21.33	157.19 ± 70.13 *	71.32 ± 20.09	3.71 ± 0.39	**0.056**	(3,36) = 2.75
**Ferritin** in µg/L	1890.80 ± 733.13 *	656.30 ± 149.31	1029.40 ± 337.89	130.00 ± 50.66	**0.033**	(3,36) = 3.24
**CRP** in µg/L	53277 ± 14763 *	38542 ± 14890	39440 ± 15520	1560 ± 361.23	**0.049**	(3,36) = 2.88
**PCT** in ng/L	0.841 ± 0.423	2.120 ± 1.935	4.332 ± 2.559	0.046 ± 0.003	0.277	(3,36) = 1.33
**D-dimer** in µg/L	2389 ± 513.44	2091 ± 406.04	2041 ± 476.70	1078 ± 258.6	0.166	(3,36) = 1.79
**PLT** in n/L	202.90 ± 25.54	227.10 ± 26.28	199.30 ± 24.43	195.90 ± 12.45	0.766	(3,36) = 0.38
**PT** in sec	12.41 ± 0.46	13.16 ± 1.47	11.60 ± 0.034	13.28 ± 1.89	0.756	(3,36) = 0.39
**INR** in (patient’s PT/control PT)	1.06 ± 0.04	1.16 ± 0.14	1.00 ± 0.03	1.22 ± 0.19	0.620	(3,36) = 0.59
**aPTT** in sec	33.13 ± 1.65	34.07 ± 6.67	34.79 ± 2.61	34.36 ± 3.43	0.992	(3,36) = 0.03
**FBG** in g/L	3.85 ± 0.35	4.51 ± 0.43	4.03 ± 0.36	3.92 ± 0.46	0.667	(3,36) = 0.52
**WBC**	9114 ± 2060	6389 ± 1073	7232 ± 1555	7791 ± 1145	0.636	(3,36) = 0.57

**Table 5:** Spearman correlations of the individuals of the *deceased* group between the blood parameters and NGF/BDNF/NFL/MMP-2/MMP-9. Significant scores are shown in bold.

	*Spearman Correlation Data*	Deceased Patients				
		NGF	BDNF	NFL	MMP-2	MMP-9
**Amylase**	SSD Rho *p*-value	156.00	88.00	194.00	125.50	118.00
		0.055	0.467	−0.176	0.239	0.285
		0.870	0.161	0.598	0.472	0.392
**Lipase**	SSD Rho *p*-value	154.00	124.00	138.00	128.50	102.00
		0.067	0.248	0.164	0.221	0.382
		0.841	0.456	0.623	0.506	0.252
**AST**	SSD Rho *p*-value	247.50	102.50	181.50	201.50	118.50
		−0.500	0.379	−0.100	−0.221	0.282
		0.133	0.255	0.764	0.506	0.397
**ALT**	SSD Rho *p*-value	255.00	202.00	110.00	262.50	205.00
		−0.545	−0.224	0.333	−0.591	−0.242
		0.101	0.501	0.317	0.076	0.467
**GGT**	SSD Rho *p*-value	250.00	162.00	152.00	226.50	216.00
		−0.515	0.018	0.079	−0.373	−0.309
		0.122	0.956	0.813	0.263	0.353
**LDH**	SSD Rho *p*-value	226.00	216.00	150.00	202.50	180.00
		−0.370	−0.309	0.091	−0.227	−0.091
		0.267	0.353	0.785	0.495	0.785
**MGB**	SSD Rho *p*-value	180.00	**32.00**	254.00	58.50	70.00
		−0.091	**0.806**	−0.539	0.645	0.576
		0.785	**0.156**	0.105	0.052	0.084
**CK**	SSD Rho *p*-value	210.00	60.00	**306.00**	**25.50**	118.00
		−0.273	0.636	**−0.855**	**0.845**	0.285
		0.413	0.056	**0.010**	**0.011**	0.392
**CK-MB**	SSD Rho *p*-value	194.00	104.50	182.00	112.50	136.00
		−0.176	0.370	−0.103	0.318	0.176
		0.598	0.267	0.757	0.339	0.598
**TNT**	SSD Rho *p*-value	293	137.00	242.00	118.00	192.00
		−0.448	0.170	−0.467	0.285	−0.164
		0.178	0.610	0.161	0.392	0.623
**IL-6**	SSD Rho *p*-value	114.00	238.00	108.00	222.50	206.00
		0.309	−0.442	0.345	−0.348	−0.248
		0.353	0.184	0.300	0.295	0.456
**Ferritin**	SSD Rho *p*-value	234.00	146.00	192.00	101.50	160.00
		−0.418	0.115	−0.164	0.385	0.030
		0.209	0.729	0.623	0.248	0.927
**CRP**	SSD Rho *p*-value	138.00	206.00	212.00	168.50	224.00
		0.164	−0.248	−0.285	−0.021	−0.358
		0.623	0.456	0.392	0.941	0.283
**PCT**	SSD Rho *p*-value	122.00	124.00	222.00	95.50	128.00
		0.261	0.248	−0.345	0.421	0.224
		0.434	0.456	0.300	0.206	0.501
**PLT**	SSD Rho *p*-value	180.00	158.00	138.00	212.50	170.00
		−0.091	0.042	0.164	−0.288	−0.030
		0.785	0.898	0.623	0.387	0.927
**PT**	SSD Rho *p*-value	107.50	161.50	118.50	189.00	111.50
		0.348	0.021	0.282	−0.145	0.324
		0.295	0.949	0.397	0.662	0.330
**INR**	SSD Rho *p*-value	188.00	174.00	100.00	171.50	114.00
		−0.139	−0.055	0.394	−0.039	0.309
		0.675	0.870	0.237	0.905	0.353
**aPTT**	SSD Rho *p*-value	150.00	156.00	176.00	141.50	146.00
		0.091	0.055	−0.067	0.142	0.115
		0.785	0.870	0.841	0.669	0.729
**FBG**	SSD Rho *p*-value	158.00	**282.00**	90.00	**238.50**	**276.00**
		0.042	**−0.709**	0.455	**−0.718**	**−0.673**
		0.898	**0.033**	0.172	**0.031**	**0.043**
**D-dimer**	SSD Rho *p*-value	200.50	231.50	154.50	171.00	215.50
		−0.215	−0.430	0.064	−0.036	−0.306
		0.518	0.226	0.848	0.913	0.358
**WBC**	SSD Rho *p*-value	122.00	196.00	114.00	225.50	206.00
		0.261	−0.188	0.309	−0.367	−0.248
		0.434	0.573	0.353	0.271	0.456

## Data Availability

Data are available on request.
